# Sleep Quality and Its Affecting Factors among Hemodialysis Patients: A Multicenter Cross-Sectional Study

**DOI:** 10.3390/healthcare11182536

**Published:** 2023-09-14

**Authors:** Bushra Alshammari, Sameer A. Alkubati, Eddieson Pasay-an, Awatif Alrasheeday, Hasna B. Alshammari, Sabah M. Asiri, Sadaa B. Alshammari, Fatimah Sayed, Norah Madkhali, Vivian Laput, Farhan Alshammari

**Affiliations:** 1 Medical Surgical Nursing Department, College of Nursing, University of Hail, Hail 2440, Saudi Arabia; s.alkubati@uoh.edu.sa; 2 Department of Nursing, Faculty of Medicine and Health Sciences, Hodeida University, Hodeida P.O. Box 3114, Yemen; 3 Maternal and Child Nursing Department, College of Nursing, University of Hail, Hail 2440, Saudi Arabia; e.pasayan@uoh.edu.sa; 4 Nursing Administration Department, College of Nursing, University of Hail, Hail 2440, Saudi Arabia; a.alrasheeday@uoh.edu.sa (A.A.); v.laput@uoh.edu.sa (V.L.); 5 Performance Improvement Unit, Hail Health Cluster, Hail 55471, Saudi Arabia; hassnaba@moh.gov.sa; 6 Health Affairs Aseer Region, Abha 62583, Saudi Arabia; sasiri9@moh.gov.sa; 7 Mawqq Primary Health Care Center, Hail Health Cluster, Hail 55471, Saudi Arabia; saadaba@moh.gov.sa; 8 Family Medicine Academy, Qassim Health Cluster, Buraidah 52367, Saudi Arabia; fosayed@moh.gov.sa; 9 Department of Nursing, College of Nursing, Jazan University, Jazan 45142, Saudi Arabia; namadkhali@jazanu.edu.sa; 10 Department of Pharmaceutics, College of Pharmacy, University of Hail, Hail 2440, Saudi Arabia; frh.alshammari@uoh.edu.sa

**Keywords:** sleep quality, hemodialysis, chronic kidney failure, Saudi Arabia

## Abstract

(1) Background: Sleep quality is one of the most important clinical outcomes of hemodialysis (HD) patients, as it can affect their physical and mental health. This study aimed to investigate sleep quality and its affecting factors among patients with HD. (2) Methods: A cross-sectional design was used to investigate the quality of sleep among HD patients in two cities in Saudi Arabia. The data were collected during the period from January to December 2022. A convenience sample of 250 HD patients was selected in this study. Data were collected using the Arabic version of the Pittsburgh Sleep Quality Index (PSQI). (3) Results: About two-thirds of participants (63.6%) had normal sleep, while one-third had poor sleep (36.4%). There was a significant relationship between the participants’ age and the quality of sleep, where participants who were aged more than 50 had poorer sleep than those who were younger (*p* < 0.001). Male and married participants significantly had poor sleep more than female and single participants (*p* = 0.011 and 0.015, respectively). In addition, participants who were retired, had a higher number of dependents, did not adhere to exercise, and had more comorbidities had significantly poorer sleep than other groups (*p* = 0.002, 0.016, 0.023, and <0.001, respectively). The level of education, financial status, distance from home to dialysis center, and participants’ satisfaction at the time of dialysis had no influence on the quality of sleep among HD patients. The multiple linear regression shows that exercise (*p* = 0.017), the number of comorbidities (*p* = 0.008), and the duration of dialysis (*p* < 0.020) were the independent factors affecting the quality of sleep among HD patients. (4) Conclusions: About one-third of HD patients in this study had poor sleep. There were significant differences between patients’ age, gender, marital status, and sleep-quality levels. Moreover, participants who retired, had a higher number of dependents, did not adhere to exercise, and had more comorbidities had significantly poorer sleep than other groups. Future studies should develop appropriate interventions to address the problem of poor sleep quality among HD patients.

## 1. Introduction

Chronic kidney disease (CKD) has become a global public health problem, and the number of people receiving renal replacement therapy is increasing rapidly annually [1]. CKD is the fourth leading cause of death among the ranked 10 most deadly diseases in the Kingdom of Saudi Arabia, and the sixteenth leading cause of death worldwide [1,2]. It is one of the most debilitating universal public health crises and the fastest growing noncommunicable disease in the United States of America [2]. It is characterized by the persistent failure of the kidney to maintain a Glomerular Filtration Rate (GFR) of no less than 60 milliliters per minute or marked Albuminuria greater than 30 milligrams per 24 h [3]. Due to a shortage of donors and appropriate medical facilities, most patients in the late stages of CKD require a kidney transplantation, or a renal replacement therapy accomplished through regular hemodialysis (HD) or peritoneal dialysis (PD) to maintain the needed body function essential for survival [4,5]. In Saudi Arabia, CKD has kept over 20,000 patients on regular dialysis, with 90% of them receiving HD [6].

Patients receiving maintenance HD experience a significant burden, due to the symptoms associated with HD treatment which affect their physical and emotional well-being [7]. There is a growing body of evidence that sleep disturbance is one of the most prevalent symptoms experienced by patients receiving HD [8,9]. Its main manifestations are easily waking from sleep, poor sleep quality, insomnia, long-term dependence on hypnotic drugs, and sleep apnea [10]. The problem of sleep quality in patients has become an important factor that threatens patients’ lives and long-term survival; increases the risk of fatigue, anxiety, memory impairment, behavioral disturbances, and depression; lowers immunity; increases the risk of cardiovascular diseases; disrupts physiologic outcomes [9,11]; and increases the risk of mortality [12]. The decline of sleep quality directly leads to the decline of the quality of life of patients receiving HD [13]. In a recent study, Mixson et al. suggested that the early identification and diagnosis of a sleep disorder among end-stage renal disease (ESRD) patients may help improve their chances of survival [12].

As for improving the patient’s sleep quality, it will help to improve mental health [14] and overall clinical outcomes [15]. Therefore, the investigation and intervention of patients’ sleep quality and its influencing factors should be paid more and more attention. The present literature is still insufficient when it comes to the documentation and discussion of sleep quality among patients receiving HD in Saudi Arabia [16]. Several studies revealed the prevalence of sleep disturbance among HD patients around the world with the incidence range 40–85% [17]. In a study that was conducted at hospitals in East Jakarta, Indonesia, more than half (56%) of HD patients were suffering from sleep disturbance [18]. In a study that was conducted for 1643 HD patients from 335 dialysis centers in US, 50% of the patients had difficulty falling asleep, 59% experienced of difficulty waking up during the night, and 49% experienced difficulty of getting up early in the morning. Overall, 53% of these patients experienced one or more of these symptoms most of the time [19]. In Saudi Arabia, a late study in 2010 was conducted by Al-Jahdali et al. that revealed 60.8% of HD patients were suffering from sleep disturbance in Riyadh and Jeddah [20].

Recent studies have also pointed out that the quality of sleep for patients who undergo regular HD is affected by many factors. A recent systematic review reported that demographics (older age and female), electrolyte imbalance, pain, and itching were associated with poor sleep quality [21]. In Turkey, poor sleep quality was observed among older patients who were unemployed and were on regular HD [22]. Sleep has always been widely accepted as one of the most critical aspects in maintaining an individual’s health and well-being. Despite this, the importance of a good night’s rest is grossly undervalued. As has been well-documented, sleep is the body’s natural way of repairing itself while recuperating lost tissues and energy [1]. In normal physiology, sleep is induced by a marked decrease in sympathetic activity and an increase in the vagal tone of the body, causing the nocturnal dipping of blood pressure. This vital process is disrupted if an individual suffers from poor sleep quality. This results in hypoxemia and sleep fragmentation, which increases sympathetic activity and decreases parasympathetic innervation, ultimately leading to a reduced fall in the nocturnal blood pressure [2].

Poor sleep quality has been associated with an increased risk of anxiety, memory impairment, and disrupted physiologic outcomes. One of the diseases that is most likely correlated with poor sleep quality is chronic kidney disease (CKD), which further impairs the quality of life for the patient. However, the present literature is still insufficient when it comes to the documentation and discussion of the relationship between sleep quality and the incidence of CKD [3]. Chronic kidney disease is the fourth leading cause of death among the ranked ten most deadly diseases in the Kingdom of Saudi Arabia, and it is the sixteenth leading cause of death worldwide [4]. It is one of the most debilitating universal public health crises and the fastest growing noncommunicable disease in the United States of America. It is characterized by the persistent failure of the kidney to maintain a Glomerular Filtration Rate (GFR) of no less than 60 milliliters per minute or marked albuminuria greater than 30 milligrams per 24 h [5].

Most patients in the late stages of CKD require a kidney transplantation or a renal replacement therapy accomplished through regular hemodialysis (HD) or peritoneal dialysis (PD) to maintain the needed body function essential for survival. In Saudi Arabia alone, CKD has kept over 20,000 patients on regular dialysis and 9810 patients on post-kidney transplant conditions [6]. Recent studies have also pointed out that the quality of sleep for patients who undergo regular HD is negatively affected. In Konya, Turkey, poor sleep quality was observed among older patients who were unemployed and were on regular HD, and one of the most important predictors of sleep quality that was noted was depression [7]. This most likely stems from the anxiety attached to aging and unemployment, coupled with the stressful environment of being admitted to the hospital for an indefinite period of time because of a chronic illness. A systematic review of sleep quality of dialysis patients pointed out that the treatment and symptom burden of dialysis disrupts and deprives patients of sleep, which leads to overwhelming and uncontrollable exhaustion [8].

Samara et al. [23] conducted a cross-sectional survey to investigate both the sleep quality and daytime sleepiness in HD patients in one of Palestine’s dialysis centers, An-Najah National University Hospital, using Pittsburgh Sleep Quality Index (PSQI) and Epworth Sleepiness Scale (ESS) [23]. The study revealed high median scores for both standardized tests, indicating poor sleep quality among HD patients. However, clinical and demographic characteristics had no significant association to the surveyed scores. Daytime sleepiness, on the other hand, was found to be less prevalent in the surveyed population.

In Saudi Arabia, insomnia was reported to be a common occurrence among HD patients and was highly related to the female gender, along with the occurrence of obstructive sleep apnea and excessive daytime sleepiness [20]. Sleep apnea was also reported to be prevalent in more than half of the patients with ESRD among the three dialysis centers in Jeddah, Saudi Arabia [24]. Patients undergoing either HD or PD were both found to suffer from recurring sleep disturbance with noted mood disorders and restless leg syndrome [25]. However, the study did not determine the factors that might influence sleep quality [25]. In view of the concluded studies regarding patients with CKD undergoing HD and its apparent effects on their sleep quality, the mentioned factors and problems in this aspect remain understudied.

By understanding the factors associated with poor sleep quality, healthcare providers, especially nurses, can assess and reinforce patients’ self-management. The findings can also guide healthcare providers to personalize appropriate sleep interventions for patients, thereby improving patients’ sleep problems. This study may help in providing the basis for further implementation of analytical research and interventional research on the maintenance of sleep-disorder strategies for hemodialysis patients in Saudi Arabia. In addition, it may increase the healthcare administrative awareness toward this problem for more investigations and solving about it.

Thus, this study aimed to explore the quality of sleep among HD patients and identify the factors affecting sleep quality in the Kingdom of Saudi Arabia.

## 2. Materials and Methods

### 2.1. Research Design

A cross-sectional design was used to investigate the quality of sleep among HD patients in two cities in Saudi Arabia. The data were collected during the period from January to December 2022.

### 2.2. Setting

This study was conducted in two dialysis centers (King Khalid Hospital and King Salman hospital) in Hail city and two dialysis centers in Al-Qassim city (Buraidah Central Hospital and DaVita Dialysis Center) in Saudi Arabia. The selected hospitals are government medical facilities for citizens that provide free services and treatment to all Saudi nationals who experience health problems. The selected hospitals were just a few of the multiple hospitals in the targeted cities, but they were the only hospitals which provide HD treatment for patients with renal failure. Each hospital can accommodate up to 500 patients in different specialties, such as oncology, cardiology, obesity, and neurology. They also include an ICU and burn, endoscopy, surgery, one-day surgery, and physiotherapy units, in addition to specialized and support service sections. A total of approximately 670 HD patients attend the selected dialysis centers each week. Each center provides HD sessions 6 days a week for approximately 80 patients each shift, which includes morning and evening: the sessions last for four hours each shift. The centers are closed one day a week. Urgent cases during the day off should go to the emergency department to receive the HD sessions.

### 2.3. Sample

A convenience sample of 250 HD patients were selected in this study. Eligible participants included those who were diagnosed with CKD stage 4 and 5 and had been receiving HD for more than three months. Participants had to be aged 18 years and older, able to read and write, and able to provide informed consent. Participants were excluded if they were not willing to participate, suffered from critical conditions, and were cognitively unable to participate. Gatekeepers, who are nursing staff in the HD centers, helped in gaining access to the study settings and participants at the centers. The gatekeepers were responsible for the process of prescreening the participants and determining whether potential participants are interested in participating in the study. Patients who fulfilled the eligibility criteria were introduced to the researcher by the gatekeepers. The researcher provided participants with the invitation letter, information sheet, and consent form. Patients were given 48 h to show interest to participate in this study. Once they agreed to participate in this study, the questionnaire was given to patients to be completed.

### 2.4. Data Collection Tools

Sociodemographic and dialysis-related information that could potentially influence the patients’ sleep experience was collected using a simple questionnaire designed by the researcher. The sociodemographic information included age, gender, marital status, occupation, level of education, number of dependents, financial situation, distance from hospital, and level of activity.

Dialysis-related participant information was also collected, including how patients are assigned to days and times in the dialysis units of the chosen hospitals. Patients were asked if they preferred to adjust their scheduled session times and whether they were satisfied with the times that had been assigned to them. The patients were also questioned if they would like a flexible dialysis schedule instead of changing the time of their dialysis sessions. The duration of dialysis and comorbidities were obtained from patients’ health records. Sleep quality levels were assessed using the Arabic version of the Pittsburgh Sleep Quality Index (PSQI). The psychometric properties of the Arabic version of the PSQI were evaluated and demonstrated adequate reliability and validity for assessing sleep quality in Arabic-speaking patients diagnosed with renal failure [26] and cancer [27]. This tool contains seven components: subjective sleep quality, sleep latency, sleep duration, habitual sleep efficiency, sleep disturbances, use of sleep medication, and daytime dysfunction. There are 19 questions in this questionnaire, with a total score ranging from 0 to 21. A lower score indicates normal quality sleep, whereas a higher score indicates low sleep quality. Participants were asked to recall their sleep status one month before enrollment and then complete 19 questions. Using the PSQI global score, patients who scored less than 5 were considered to have normal sleep, and patients who scored 5 or more were considered to have poor sleep.

### 2.5. Ethical Considerations

Ethical approvals were obtained from the research ethics committees at University of Hail number, Institutional Review Board committee at Hail health clusters, and General directorate of health affairs in Al-Qassim city (Approval Nos: (H-2021-206), (H-08-L-074), and (607-44-2091), respectively). During data collection, head nurses in the intended centers assisted in presenting a list of the possible participants who met the appropriate criteria and showed interest in participating in the study. After introducing themselves to the patients, the researchers went over the goals and design of the study. Prior to the study’s procedures, each subject gave his/her written informed consent. Participants were made aware that their participation in the study was entirely voluntary and that they might leave at any time. The quiz took no more than 10 min to complete.

### 2.6. Data Analysis

Data were analyzed using the IBM SPSS Statistics software, Version 27 (IBM Corp., Armonk, NY, USA). Mean and standard deviation (SD) were used to represent normally distributed continuous variables, whereas frequencies and percentages were used to describe normally distributed categorical variables. The chi-square test was used to determine the relationship between the independent variables and the quality of sleep. Binary linear regression was used to further evaluate factors that were significantly linked to participants’ quality of sleep. Statistical significance was set at *p* < 0.05.

## 3. Results

Table 1 illustrates that most of the participants were aged more than 50 years (43.6%), with a mean ± SD of (49.87 ± 16.13). More than half of them were male (57.6%), and the majority was married (82.4%). Regarding their work situation, more than one-third (38.8%) were unemployed, followed by employed and retired (29.2% and 26%, respectively). Approximately, less than half of participants (45.2%) had secondary school, and less than one-quarter (24%) of them were illiterate. Around one-third had 4–6 dependents (32.8%), followed by 1–3 and ≥7 at 30.8% and 23.6%, respectively. The majority had an acceptable level of financial status and did not provide exercise in their life (72.8% and 88.4%, respectively). The dialysis center was approximately 20–15 min away for less than half of the participants (44%), and less for than half (46%), their dialysis duration was 4 h or more. The majority of participants (96%) was satisfied with the time of dialysis, with no preference to change the time (86%). Finally, around half of the participants had selected the time themselves (48%).

As shown in Table 1, there was a significant relationship between the participants’ age and the quality of sleep, as participants who were aged more than 50 had poorer sleep than those who were younger (*p* < 0.001). Male and married participants had significantly poorer sleep more than female and single participants (*p* = 0.011 and 0.015, respectively). In addition, participants who retired, had a higher number of dependents, did not adhere to exercise, and had more comorbidities had significantly worse fatigue than other groups (*p* = 0.002, 0.016, 0.023, and <0.001, respectively). On the other hand, there was no significant relationship between the quality of sleep and the items of level of education, financial status, distance of home from dialysis center, satisfaction with the time of dialysis, and who selected the time of dialysis (*p* > 0.05).

**Figure 1:**
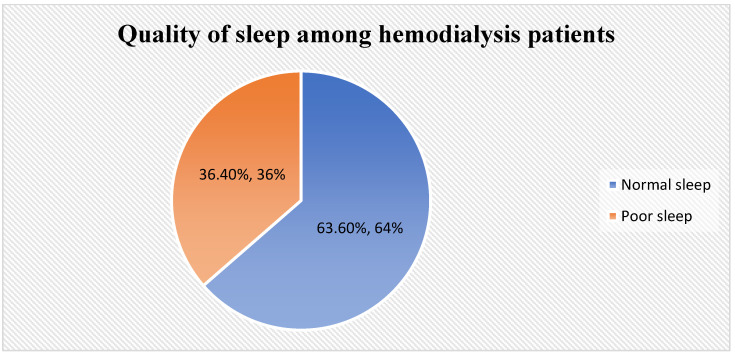
Quality of sleep among HD patients.

Figure 1 illustrates that about two-thirds of participants (63.6%) had normal sleep, while one-third had poor sleep (36.4%).

The multiple linear regression shows that exercise (*p* = 0.017), number of comorbidities (*p* = 0.008), and the duration of hospitalization (*p* < 0.020) were the independent factors affecting the quality of sleep among HD patients (Table 2).

## 4. Discussion

This study aimed to investigate the quality of sleep and its associated factors among HD patients in Saudi Arabia. In this current study, poor sleep does occur in HD patients, but it was only noted in one-third of patients, and these disturbances have been linked to a variety of negative health outcomes, including depression, anxiety, and a lower quality of life. For example, sleep disorders can impair treatment adherence in hemodialysis patients. Moreover, interrupted sleep can result in missed dialysis sessions, medication non-compliance, and decreased engagement in their overall treatment plan [28]. As shown in several studies [29,30], HD patients may experience a variety of sleep problems, including trouble falling asleep, rising frequently throughout the night, waking up early in the morning, and poor sleep quality overall. While the present finding may differ from that of the earlier studies, this can be explained by a variety of factors, including the study population’s individual features, methodology used, and variances in healthcare practices. Variations in the prevalence rates of sleep disturbances can be noticed when comparing different research that explores sleep quality in hemodialysis (HD) patients.

The poor sleep quality reduces HD patients’ quality of life greatly. In other words, patients’ capacity to participate in daily activities, enjoy social relationships, and retain a sense of well-being may be severely hampered. This deterioration in quality of life can result in feelings of dissatisfaction, powerlessness, and diminished independence. There are a number of potential causes of sleep disturbances, including renal disease itself and the dialysis procedure. Fluid overload, restless legs syndrome, medication side effects, anxiety, sadness, and the disruptive nature of the dialysis schedule are all possible causes of poor sleep in HD patients [30,31,32,33]. According to Elias and colleagues [34], HD patients may retain excess fluid, which can cause pain, breathing difficulties, and frequent nighttime awakenings due to the need to urinate. Typically, restless leg syndrome worsens during periods of rest or inactivity, resulting in sleep disturbances and decreased sleep duration. Furthermore, it is well recognized that drugs regularly used in HD patients can have sleep-related side effects. Furthermore, anxiety might aggravate pre-existing sleep difficulties. Dialysis session timing and frequency can interrupt the natural sleep–wake cycle. HD patients frequently have to accommodate dialysis sessions in the early morning or late at night, resulting in inconsistent sleep patterns [34].

As shown in numerous studies, some of the options that healthcare providers have at their disposal in order to address sleep issues in HD patients include optimizing dialysis treatment parameters, managing fluid balance, addressing comorbid conditions, promoting sleep hygiene practices, and considering the use of pharmacological interventions if necessary [35,36,37]. However, treatment methods should be adapted to the specific requirements of the patient, as well as the underlying causes of the individual’s sleep disorders [38]. For instance, individualizing the dialysis treatment plan for patients with fluid-overload-related sleep problems can be done to address fluid removal, while avoiding fast shifts that could cause hypotension. Moreover, the healthcare providers should consider changing the frequency and duration of dialysis treatments based on the patient’s residual kidney function and fluid intake to optimize fluid balance. There was a significant relationship between the participants’ age and the quality of sleep where participants who were aged more than 50 had poorer sleep than those younger age. This implies that it appears that there may be a correlation between increasing age and an increased risk of experiencing sleep issues among HD patients [32]. It is essential to keep in mind the possibility that this relationship is influenced by a variety of different circumstances.

According to Crowley (2011), older people frequently encounter unique issues and anxieties that can disrupt their sleep patterns. These difficulties are caused by both physiological changes associated with aging and a variety of extrinsic circumstances. For example, people’s sleep architecture changes as they age, with a decrease in deep sleep (slow-wave sleep) and a greater proportion of lighter sleep stages. This can result in more frequent nighttime awakenings and interrupted sleep. It is essential to keep in mind the possibility that this relationship is influenced by a variety of different circumstances [39]. Chronic renal disease and the requirement to undergo HD can cause a person to experience increased physical discomfort, disturbances in the natural sleep–wake cycle, and increased psychological distress [33,40]. In addition, people who are older may be more likely to experience changes in their sleep that are associated with aging, such as a decrease in the quality of their sleep and an increase in the prevalence of sleep disorders [41,42].

Meanwhile, in this study, the male and married participants had significantly poorer sleep more than the female and single participants, meaning that there is a significant gender and marital-status disparity in HD patients on sleep patterns. While we cannot find direct works from the literature to explain this finding, scholars such as Eldridge-Smith and colleagues [43] have explained that, among married men, increased perceived household responsibility was associated with greater sleep disturbances. Male and marriage participants are more likely to be exposed to more stress related to their responsibilities toward their families that affects their sleep [44]. They may be more worried about their health and the well-being of their families, which can increase their stress levels and make it difficult to fall asleep [45]. Sleep apnea, which can result in disturbed sleep, may affect males more frequently than it does females [46]. Melatonin is a hormone that plays a role in the regulation of sleep, and males often have lower quantities of this hormone than females [47]. While hormone levels do play a role in male–female sleep disparities, the overall picture is complex and multifaceted. Disparities in sleep are caused by a combination of hormonal, metabolic, psychological, social, and environmental factors. Understanding these complexities is critical for designing effective therapies and treatments for sleep problems that take into account patients’ overall well-being [48].

In addition, participants who were retired, had a greater number of dependents, did not adhere to exercise, and had more comorbidities had significantly higher fatigue than other groups. Such results mean that patients on HD who are retired, have a larger number of dependents, do not consistently engage in physical activity, and have a greater number of comorbidities report much higher degrees of fatigue compared to patients in other patient groups. Due to the fact that these patients are retired, it is possible that they do not participate in regular employment activities, which may result in a lifestyle that is more sedentary. According to Bai and associates’ [49] study, fatigue can be caused by a lack of physical exercise, as well as reduced involvement in activities. Moreover, patients who do not follow their prescribed exercise regimens run the risk of experiencing a reduction in their physical conditioning, which can contribute to an increase in fatigue [50,51,52,53]. It is possible that these patients have more responsibilities and obligations, such as taking care of members of their own families, because they have a greater number of dependents [54]. These obligations can lead to higher levels of stress and demand more physical and emotional energy, both of which can contribute to increased levels of fatigue.

Patients undergoing HD frequently suffer from a number of comorbid disorders, including diabetes, hypertension, cardiovascular disease, and other persistent illnesses [55,56]. As shown in Jacobson and partners’ [57] study, a higher total symptom burden can be caused by the existence of several extra health conditions, which can also make fatigue worse. In order to guide the assessment process, nurses must have a firm grasp on the specific elements contributing to fatigue, such as retirement, dependents, exercise adherence, and comorbidities. Nurses can help patients and their families understand the benefits of staying active throughout life, including retirement. Promoting the value of regular exercise and providing suggestions for incorporating exercise into daily life. Nurses can also assist patients emotionally and look for ways to help them become more responsible. One way to do this is to help them find local support networks or professional help, such as counseling.

Conversely, there was no significant relationship between the quality of sleep and the items of level of education, financial status, distance of home from dialysis center, participants’ satisfaction of the time of dialysis, and who selects the time of dialysis. These results mean that other factors, such as the underlying medical condition, the medications that are being used, the stress of living with chronic kidney disease, issues related to lifestyle, comorbidities, or the state of mental health are more important drivers of the quality of sleep that HD patients obtain. While education and financial position are not the key drivers of sleep quality in HD patients, differences in these areas can nevertheless have an impact on overall health outcomes and well-being. The specific problems of managing kidney illness and receiving hemodialysis treatment can create a more nuanced picture in which factors other than education and socioeconomic status may have a greater impact on sleep quality. This finding is in accordance with the studies of Mujahid and colleagues [58] and Firoz and associates [32], who specifically mention that they have not found a significant relationship between the quality of sleep and level of education among HD patients.

Moreover, several studies show that there is no significant relationship between the quality of sleep and financial status among HD patients [58,59]. Furthermore, studies with varying methodologies and patient populations have reached the same conclusion: there is no correlation between HD patients’ sleep quality, their level of satisfaction with their dialysis schedules, or the identity of the person making these decisions [60,61]. The lack of correlation between these variables and sleep quality suggests that nurses should instead concentrate on providing comprehensive interventions that target a wide range of sleep-related issues. This may involve managing comorbidities, such pain or anxiety, that may impair sleep; promoting a regular sleep pattern; reducing sleep disturbances during dialysis sessions; and educating on relaxing techniques or sleep-promoting measures. Educating patients on the value of sleep and how it affects their health and well-being should be a top priority for nurses. HD patients have complicated needs, and nurses should interact with other healthcare experts, such as nephrologists, dietitians, psychologists, and social workers, to meet those needs. By working together, providers may give patients the care they need regardless of what may be causing their sleep problems, such as nutritional deficiencies, emotional anguish, or a lack of social connections.

The number of comorbidities and duration of hospitalization were factors affecting the quality of sleep among HD patients. This means that the quality of sleep can be affected by factors such as the presence of various comorbidities, as well as the length of time that HD patients were hospitalized. Comorbidities such as hypertension, diabetes, cardiovascular disease, and kidney-related problems are common among patients on maintenance HD and peritoneal dialysis and might have an indirect or direct effect on sleep quality [31,58,62,63]. Poor sleep quality is common among dialysis patients, and studies have shown that being on HD and having a mental condition are both risk factors [64]. Regardless of the type of dialysis used, patients have been found to have significantly poorer sleep quality compared to healthy controls of the same age and gender [63]. The sleep quality of HD patients was shown to be quite poor in a multicenter research study, with 66.4% of patients having a PSQI > 5 [62]. HD patients who suffer from chronic discomfort are more likely to experience psychological distress, such as insomnia and depression, and may be more likely to consider discontinuing treatment [65].

HD patients may experience poor sleep quality and sleep disruptions due to their prolonged hospitalization [13,17,66]. There is a significant weight of physical and mental symptoms experienced by HD patients which has a direct impact on their ability to sleep and their overall quality of life [63]. As shown in Lin et al.’s [13] study, among patients undergoing maintenance HD, 61.78 percent reported experiencing some form of sleep disturbance. Moreover, the authors mentioned that several modifiable characteristics were also found which will aid clinical caregivers in developing individualized sleep hygiene therapies to help patients with sleep disorders. Patients with renal failure who are treated with modalities other than maintenance HD may have longer total sleep time, better sleep efficiency, and less sleep fragmentation [17]. HD patients often suffer from severe insomnia, which may be exacerbated by the disruption of normal sleep/wake cycles caused by treatment and scheduling complications [66]. It is critical for nurses to evaluate and address these issues in order to enhance HD patients’ sleep hygiene and health. The detrimental impact on sleep quality in this at-risk population can be mitigated by the implementation of interventions, such as patient education on sleep hygiene practices, providing a pleasant and quiet setting, and partnering with the healthcare team to successfully manage comorbidities.

One strength of our study is that it was conducted in two centers of two governorates in Saudi Arabia. However, there are some limitations. First, this study used a cross-sectional and quantitative approach; a longitudinal and qualitative study is recommended to explore more details of the phenomenon. Second it used a convenience sample that may lead to risk bias and decrease the representative of the studied population. Gatekeepers could also limit access to certain patients. Another limitation is that some factors were not included in this study, such as uremia itching, and need to be included in future studies.

## 5. Implication to Nursing Practice

The high frequency of poor sleep quality and insomnia among HD patients in Saudi Arabia presents a significant challenge for the country’s healthcare system. It is crucial for nurses to understand the causes of poor sleep quality in HD patients and the high prevalence of sleep disruptions in this population. Nurses can improve sleep quality by using individualized therapies after considering issues such as pain, depression, anxiety, and insufficient dialysis adequacy. Patients’ health and well-being can benefit from routine sleep assessments as part of nursing care [3], as well as from the implementation of evidence-based treatments to improve sleep hygiene and alleviate the symptoms associated with insufficient sleep [53]. Nurses in Saudi Arabia can help improve the quality of care given to HD patients by addressing sleep disorders. Overall, understanding the consequences of sleep quality and its influencing factors in hemodialysis (HD) patients is critical for clinical practice and patient well-being. The link between sleep quality and HD provides distinct problems that healthcare providers must address in order to maximize patient care. Recognizing sleep quality as an important component of overall health is critical. Healthcare practitioners must take a comprehensive approach to patient treatment that incorporates not just the physical aspects of HD but also the psychological and emotional well-being of patients. They need to include regular sleep quality assessments in the normal care practice for HD patients and use proven techniques to measure sleep patterns, disruptions, and daytime drowsiness. This information can be used to suggest customized interventions. Incorporating sleep-focused care into the HD patient’s experience can improve overall well-being, treatment adherence, and quality of life. Healthcare practitioners can make a substantial contribution to the holistic care of HD patients by addressing sleep problems and their underlying variables.

## 6. Conclusions

About one-third of HD patients in this study had poor sleep. There were significant differences between age, gender, married patients, and sleep quality. Moreover, participants who were retired, had a greater number of dependents, did not adhere to exercise, and had more comorbidities had significantly greater fatigue than other groups. Conversely, there was no significant relationship between the quality of sleep and the level of education, financial status, distance of home from dialysis center, participant’s satisfaction at the time of dialysis, and who selects the time of dialysis. Lastly, the exercise, number of comorbidities, and duration of hospitalization were the factors associated with the quality of sleep among HD patients. Nurses play a critical role in recognizing, diagnosing, and managing sleep disorders in order to promote overall patient well-being. They assess patients to detect sleep abnormalities, for example, during routine checkups or dialysis treatments. Moreover, they use proven sleep assessment instruments to collect extensive information about sleep patterns, sleep quality, and daytime drowsiness. They also can provide information about the importance of sleep and its impact on overall health. It is important that they educate patients on common sleep problems, their symptoms, and possible causes. Therefore, it is recommended that nurses contribute to improving the quality of life of these patients through the provision of education, collaboration with other members of the healthcare team, and monitoring of the success of interventions.

## Figures and Tables

**Table 1:** Relationship between participants’ sociodemographic characteristics and their sleep quality.

Variable		N (%)	Normal Sleep	Poor Sleep	*p*-Value
Age	Less than 35	53 (21.2)	39 (24.5%)	14 (15.4%)	<0.001
	35–50	88 (35.2)	68 (42.8%)	19 (22.0%)	
	More than 50	109 (43.6)	52 (32.7%)	55 (62.6%)	
Sex	Male	144 (57.6)	82 (51.6%)	62 (68.1%)	0.011
	Female	106 (42.4)	77 (48.4%)	29 (31.9%)	
Marital status	Married	206 (82.4)	124 (78.0%)	82 (90.1%)	0.015
	Single	44 (17.6)	35 (22.0%)	9 (9.9%)	
Occupational status	Employed	73 (29.2)	46 (28.9%)	27 (29.7%)	0.002
	Unemployed	97 (38.8)	70 (44.0%)	27 (29.7%)	
	Student	15 (6.0)	13 (8.2%)	2 (2.2%)	
	Retired	65 (26.0)	30 (18.9%)	35 (38.5%)	
Level of education	Illiterate	60 (24.0)	36 (22.6%)	24 (26.4%)	
	Primary	30 (12.0)	18 (11.3%)	12 (13.2%)	0.811
	Secondary	113 (45.2)	73 (45.9%)	40 (44.0%)	
	Higher education	47 (18.8)	32 (20.1%)	15 (16.5%)	
Number of dependent	No dependent	32 (12.8)	28 (17.6%)	4 (4.4%)	0.016
	1–3	77 (30.8)	49 (30.8%)	28 (30.8%)	
	4–6	82 (32.8)	50 (31.4%)	32 (35.2%)	
	≥7	59 (23.6)	32 (20.1%)	27 (29.7%)	
Financial status	Excellent	48 (19.2)	26 (16.4%)	22 (24.2%)	0.214
	Acceptable	182 (72.8)	118 (74.2%)	64 (70.3%)	
	Bad	20 (8.0)	15 (9.4%)	5 (5.5%)	
Exercise	Yes	29 (11.6)	24 (15.1%)	5 (5.5%)	0.023
	No	221 (88.4)	135 (84.9%)	86 (94.5%)	
Number of Comorbidities	No comorbidity	66 (26.4)	56 (35.2%)	10 (11.0%)	<0.001
	1	29 (11.6)	14 (8.8%)	15 (16.5%)	
	2	72 (28.8)	46 (28.9%)	26 (28.6%)	
	≥3	83 (33.2)	43 (27.0%)	40 (44.0%)	
Distance	Less than 10	51 (20.4)	33 (20.8%)	18 (19.8%)	0.149
	10–15	110 (44.0)	77 (48.4%)	33 (36.3%)	
	16–20	57 (22.8)	33 (20.8%)	24 (26.4%)	
	More than 20	32 (12.8)	16 (10.1%)	16 (17.6%)	
Dialysis duration	One or less	60 (24.0)	41 (25.8%)	19 (20.9%)	0.014
	2–3	74 (29.6)	55 (34.6%)	19 (20.9%)	
	4 or more	116 (46.4)	63 (39.6%)	53 (58.2%)	
Satisfaction	Satisfied	240 (96.0)	155 (97.5%)	85 (93.4%)	0.113
	Not satisfied	10 (4.0)	4 (2.5%)	6 (6.6%)	
Preference to change time	Yes	16 (6.4)	12 (7.5%)	4 (4.4%)	0.030
	No	215 (86.0)	140 (88.1%)	75 (82.4%)	
	Sometimes	19 (7.6)	7 (4.4%)	12 (13.2%)	
Who select time	Patient	120 (48.0)	81 (50.9%)	39 (42.9%)	0.218
	Not the patient	130 (52.0)	78 (49.1%)	52 (57.1%)	

**Table 2:** Predictors of factors affecting quality of sleep among HD patients.

Factor		B	S.E.	Wald	Sig.	Odds Ratio (95% C.I.)
Age		0.010	0.016	0.411	0.522	1.010 (0.980–1.041)
Sex	Male	Ref				
	Female	0.003	0.451	0.000	0.994	1.003 (0.415–2.427)
Marital status	Married	Ref				
	Single	−0.608	0.518	1.380	0.240	0.544 (0.197–1.501)
Occupational status	Employed	Ref				
	Student	−0.145	1.032	0.020	0.889	0.865 (0.114–6.541)
	Retired	0.143	0.456	0.098	0.754	1.154 (0.472–2.818)
	Unemployed	−0.486	0.482	1.017	0.313	0.615 (0.239–1.582)
Number of dependents		0.024	0.066	0.136	0.713	1.025 (0.900–1.167)
Exercise	Yes	Ref				
	No	1.506	0.631	5.696	0.017	4.509 (1.309–15.535)
Number of comorbidities	No	Ref				
	1	1.151	0.482	5.693	0.020	3.161 (1.228–8.136)
	2	0.383	0.400	0.919	0.338	1.467 (0.670–3.212)
Duration of dialysis months		0.000	0.003	0.002	0.968	1.000 (0.995–1.005)
Preference to change time	Yes	Ref				
	No	0.236	0.665	0.126	0.723	1.266 (0.344–4.665)
	Sometimes	1.391	0.804	2.996	0.083	4.018 (0.832–19.407)

B, coefficient of predictor variables; C.I., confidence interval; S.E., standard error.

## Data Availability

The data presented in this study are available on request from the corresponding author.
